# Factors within A Veterinarian-Cattle Farmer Relationship That May Impact on Biosecurity Being Carried out on Farms: An Exploratory Study

**DOI:** 10.3390/vetsci10070410

**Published:** 2023-06-23

**Authors:** Nikisha Grant, Heather Buchanan, Marnie L. Brennan

**Affiliations:** 1 School of Medicine, University of Nottingham, Queen’s Medical Centre, Nottingham NG7 2UH, UK; nikisha.grant@adelphigroup.com (N.G.); heather.buchanan@nottingham.ac.uk (H.B.); 2 School of Veterinary Medicine and Science, University of Nottingham, Sutton Bonington Campus, Loughborough LE12 5RD, UK

**Keywords:** biosecurity, veterinarian, vet, farmer, cattle, relationship, communication, thematic analysis, interviews

## Abstract

**Simple Summary:**

There is much research work describing how important the veterinarian–farmer relationship is to the decisions that are made by farmers. There is less information about how features of the veterinarian–farmer relationship could potentially affect how and whether biosecurity (prevention and control measures) is carried out. The aim of this study was to explore factors within the veterinarian–cattle farmer relationship that could influence the likelihood of biosecurity measures being taken on cattle farms in England. Cattle farmers and veterinarians carrying out high levels of biosecurity were interviewed, with a focus on exploring communication and the perceived influence each had on the other. Five interconnecting themes were identified, focusing on issues of trust, time, getting to know each other, the ability to have cooperative discussions and clarification regarding the cost-effectiveness of measures. It appears that this relationship and potentially how these interactions occur are likely to be critical to any future disease-prevention planning and implementation efforts.

**Abstract:**

Veterinarians (vets) appear to be one of the main gateways to biosecurity information for cattle farmers, and therefore are likely to affect the implementation of these measures. The aim of this study was to explore factors within the vet–farmer relationship that may impact on biosecurity being carried out on cattle farms in England. Interviews were conducted with cattle farmers and large-animal vets, with a focus on individuals deemed to implement good levels of biosecurity or those working with said individuals. The questions explored how each stakeholder felt the communication occurred between the groups and the perceived consequential influence each had on the other. Inductive Thematic analysis was used to explore participants’ experience of vet–farmer interactions with a focus on areas of reciprocity between the two groups. Five primary themes were identified. Factors within the vet–cattle farmer relationship, such as trust and familiarity, which were interconnected with time spent with each other, appeared to influence the uptake of biosecurity measures on cattle farms. These factors purportedly impacted the ability of vet–farmer pairs to have cooperative discussions and enter into shared decision-making. In order to enhance animal and human health and welfare, these relationship factors might be key to the development of sustainable optimisation frameworks.

## 1. Introduction

“Biosecurity” has been defined as practices that reduce the potential for the introduction or transmission of organisms onto and/or between farms that cause disease in animal. These practices benefit the health of farm animals and the upkeep of the farm itself. Examples of important biosecurity practices could be purchasing animals from a certified disease-free herd, restricting access to animals to only those individuals or businesses playing a key role (e.g., veterinarians) within a farming system, and disinfecting or changing protective clothing between handling different animal management groups. The importance of biosecurity was emphasised in the UK during the outbreak of foot and mouth disease (FMD) in 2001. This saw the disease infect farm animals across the UK within a matter of weeks, causing roughly six million farm animals to be slaughtered [1] and leading Britain into the largest agricultural crisis it has ever seen. Since this devastation, steps have been taken to attempt to avoid another catastrophic outbreak in the future [2]. Despite the increased research and awareness around biosecurity, farms around the UK continue to vary considerably in their preventative practices [3,4], indicating that the hygiene of farm animals also varies substantially. This is not only a concern for animal welfare, but directly impacts human health, as biosecurity is critical for maintaining food standards and preventing human-harming diseases [4], such as campylobacteriosis [5] and cryptosporidiosis [6]. Lower disease levels amongst cattle leads to outcomes such as lower veterinarian (vet) costs, higher calf birth rates, fewer cattle deaths and, in turn, less purchasing of replacement cows [7], all of which contribute to increased profit margins that benefit farmers [4]. Additionally, as antimicrobial resistance is one of the major threats to both animal and human health worldwide, healthier animals mean less use of antimicrobials, leading to potentially fewer problems with antimicrobial resistance [8].

While research has increased the theoretical knowledge around biosecurity measures, less attention has been paid to the practical implementation of these preventative practices. The implementation of biosecurity measures primarily involves a collaboration between a farmer and their vet to formulate feasible measures to put into place on a cattle farm. Although it has been reported that many factors influence farmers’ decision-making [9], vets are often seen as an influential and trusted source of biosecurity information for farmers [10,11,12,13,14,15]. This fits alongside previous research that indicates that targeted and bespoke recommendations from individuals who are familiar with the specific local context or conditions are more likely to be followed [16,17,18]. However, not only is there currently minimal research into vet and cattle farmer communication and relationships, the available evidence suggests that vet–farmer relationships are often unbalanced and strained [14,19,20]. This can cause biosecurity discussions to be ineffective and the subsequent biosecurity practices to be unsuccessful [21], resulting in a myriad of problems. These tensions within vet–farmer relationships can be accentuated by some vets taking a paternalistic role during consultations [21], and not co-producing sustainable solutions for known issues, including biosecurity planning [22]. Consequently, farmers can be left confused and sidelined by these discussions and are uninspired to implement sufficient biosecurity measures on their farms [19,23]. More recently, work has been conducted using structured tools to assess the adequacy of vet–farmer communication [24], with a focus on training vets in specific communication techniques [25,26]. However, further research is needed, using a bottom-up exploratory perspective [27], which could provide further insight from both cattle farmers and large-animal vets on the type of communication required to generate real change. 

It is possible that this vet–farmer ‘divide’ in relation to communication could be informed by wider human healthcare research, as described in Ritter, Adams, Kelton and Barkema [24]. Medical research shows that, within doctor–patient consultations, a shared understanding of the medical issue at hand [28], good rapport between patient and clinician [29], quality information exchange [30] and shared decision-making for medical treatments [31] are integral to effective communication and a strong relationship between doctors and patients. It is of interest to assess whether these factors are similar for vets and farmers in relation to what constitutes good communication and the impact of the communication on the uptake of recommendations.

Therefore, the aim of this study was to explore the factors within the vet–cattle farmer relationship that could have an impact on the level of biosecurity being carried out on cattle farms in England. 

## 2. Materials and Methods

Sampling: Cattle farmers and large-animal vet surgeons based in the UK were recruited for interviews using convenience sampling. Participants who were previously involved in biosecurity studies [11] were approached, as well as advertisements being placed on the personal social media sites (Twitter and/or Facebook) of the first and last authors, and the Twitter and Facebook sites of the Centre for Evidence-based Veterinary Medicine, to recruit additional participants. Individuals that expressed an interest in the study were sent an information sheet (containing details about what the study would entail) and a consent form (addressing their involvement and the fact the interview would be audio-recorded) via email, which was to be completed before the interview took place. The researchers aimed to recruit 10 vets and 10 cattle farmers as a maximum, dictated by the time and resource available, or if it was deemed that adequate data richness and complexity to address the research question had been achieved, this was denoted as data saturation [32]. ‘Meaning saturation’ was reached before the maximum number of participants was achieved, as new incoming data provided no new information in relation to the research question [33]. Therefore, interviews ceased and an analysis was conducted on the themes that were repeatedly present in the data. ‘Meaning saturation’, as a technique guiding qualitative analysis, has been outlined in previous research papers [33]. 

Inclusion criteria: Farmers were eligible for the study if they owned a herd of cattle and were self-certified as having good levels of biosecurity on their farm (see ‘Materials’ section below for further detail). Vets were eligible if they were working with at least one farmer and one herd of cattle who had good levels of biosecurity. These criteria were confirmed over email prior to the interviews taking place. By only interviewing participants who with perceived ‘good’ levels of biosecurity, any common themes generated from the interviews had implied importance in the implementation of the biosecurity measures being undertaken, regardless of whether the themes were related to positive or negative aspects of the relationship. No incentives were offered to participate in the research.

Data collection process: Qualitative data were collected using semi-structured interviews that took place over the telephone, either using “Skype” or face-to-face (all interviews were audio-recorded). The interviewer for all interviews was the female first author (a Masters’ of Science student in Health Psychology at the time of the study and a BSc Psychology graduate). The interviewer received training on study design and the execution of qualitative research methodology and data analysis as part of her MSc training. Although participants had already provided written consent to take part in the study, interviews began by the participant verbally confirming their consent to be involved, before the interviewer offered some brief information about her own research background, a recap of the study’s background and aims, and an overview of what the interview would entail.

The interview guide developed by the first author was discussed with the supervisory team (HB, MB) and members of the Centre for Evidence-based Veterinary Medicine, University of Nottingham, research team as part of a pre-testing process to optimise the clarity of the questions being asked. The interview schedule and process were piloted by one vet and one farmer, with both vet and farmer interview questions deemed appropriately clear and understandable to achieve the aims of the study.

Materials: Separate semi-structured interview schedules were developed for cattle farmers and for vets (Supplementary Materials) so that their personal experience of biosecurity and their vet–farmer relationship could be specifically explored. The interview schedule involved open and closed questions. Initially, the participant was asked about their experience as a vet/cattle farmer. They were then requested to hold a certain cattle farmer/vet in mind for the duration of the interview. Participants were then asked about the biosecurity on their/the chosen farm, as this allowed for the interviewer to identify the standard of biosecurity being carried out. The interviewer’s knowledge about biosecurity was gained from their background research, discussions with the final author (experienced researcher in this area) and during the pre-testing phase of the interview questions with the veterinary researcher team from the Centre for Evidence-based Veterinary Medicine. The level of biosecurity was judged by the researcher, which depended on how the participant described biosecurity and what measures were being undertaken on the cattle farm. If levels were deemed insufficient by the researcher, the participant’s transcript would be analysed separately from the main data analysis. Questions addressing the biosecurity discussions that had previously taken place with their farmer/vet, and what they personally believed motivated them/their cattle farmers to implement good biosecurity were asked. Interviews were recorded on a mobile device and transcribed verbatim onto a computer immediately after the interview by the first author. In order to maintain participant confidentiality and anonymity, none of the participant’s identifiable details were transcribed.

Data analysis: Inductive Thematic Analysis (TA) [32] was chosen as a suitable analysis method. An essentialist epistemological approach to TA [32] was deemed most appropriate, as the study aimed to explore participants’ direct experience of their vet–farmer consultations and of biosecurity being carried out. Thematic Analysis allows for researchers to assess rich qualitative data by assigning concepts to codes and then gathering similar related codes into themes that summarise participant responses [32]. The resulting themes were reviewed and named as finalised themes, which were likely to represent factors deemed important in vet–farmer relationships that could impact the biosecurity being undertaken on cattle farms. The following six steps of thematic analysis were undertaken according to the methods outlined by Braun and Clarke [32]:

1. Familiarisation with the data;
2. Generation of initial codes;
3. Identification of themes;
4. Reviewing themes;
5. Definition and naming of themes;
6. Final construction of results.

The first author carried out inductive TA on all participant transcripts by hand, without the use of software, evaluating transcripts using the participants’ verbalised language rather than interpreting responses by searching for underlying latent meanings. Firstly, all vet transcripts were thoroughly read through a number of times and the key factors and topics derived from each response to a question were noted. Reciprocal factors, or factors that participants felt strongly about, regarding their vet/farmer relationship, vet/farmer communication and biosecurity, were noted. These were then compared across all vet transcript texts in order to synthesise identified factors into codes. The codes were discussed with the middle author. Once the initial codes were identified within the texts, they were organised in terms of their relatability to each other in order to produce themes. After themes were refined and finalised, all vet transcripts were read through for a final time to confirm that no context or important features were lost during the analysis process. This procedure was then repeated for the farmers’ transcripts, and for a third time with all transcripts contrasted together. This resulted in prominent themes from vet interviews, farmer interviews, and from both participant groups collectively.

This study was reported using the suggested structure within the COREQ reporting guidelines [34]. The research was reviewed and approved by the University of Nottingham (UK) Rehabilitation & Ageing Divisional ethics review committee (reference number RPI-18-03) prior to the commencement of the study.

## 3. Results

Interviews were conducted between June and August 2018. In total, nine vets and eight cattle farmers were interviewed. This included seven male and ten female participants, aged between 24 and 60 years old. Interviews lasted an average of 20 min (range 5 min–1 h) and there was no difference between the average length of interview between vets and farmers. It was deemed that data saturation [33] to address the research question was achieved after 17 interviews, so data collection ceased at this point. Two of the seventeen interviews were conducted in person (both farmers in their homes), one was conducted via Skype and the remaining fourteen were carried out over the telephone. A mixture of beef and dairy farmers were interviewed, with a median herd size of 115 (interquartile range IQR 36–228). Farmers had been farming for a median of 13 years (IQR 9–40) and were primarily based in the Midlands and the south of England. At least one farmer ran an organic farming operation. Vets had been practicing for a median of 10.5 years (IQR 9.75–11.25) and were located across the UK from southern England to the south of Scotland. They saw the farmer in question primarily on a weekly or fortnightly basis and had been that farmer’s vet for a median of 3 years (IQR 8 months–6 years), with at least one running an organic farming operation.

During one of the interviews, one vet stated that their farmer was not carrying out sufficient levels of biosecurity. In response, this transcript was analysed separately from the rest of the transcripts to avoid divergence from the analysis results. However, as this interview generated similar concepts to those from the other interactions, it was deemed appropriate to analyse the data from all interviews together.

**Figure 1:**
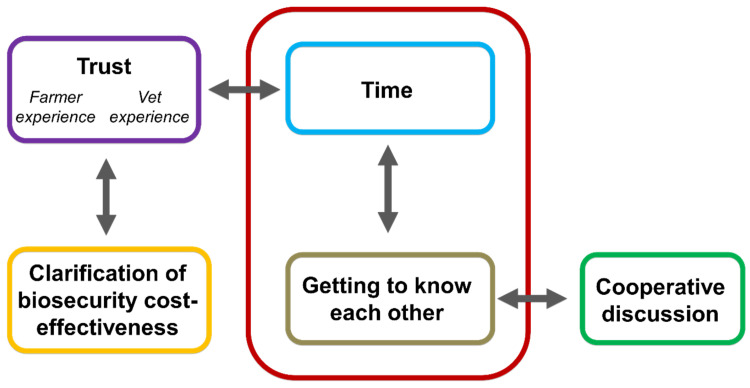
Finalised themes (represented by 5 individual-coloured boxes) deemed to be important factors within a vet–farmer relationship that may impact biosecurity on farms. The large red box indicates significant interpretive interactions between themes; grey arrows indicate interpretive interactions between themes (see text for further details).

Thematic analysis led to a total of 46 codes being identified. Five overarching themes were ascertained (Figure 1): trust, time, getting to know each other, clarification of biosecurity cost-effectiveness and cooperative discussion. Trust encompassed two sub-themes (cattle farmer and vet experience). The interpretation of these themes and the possible interactions between them make presenting them in a single linear format challenging; they are therefore presented in the order that makes the most logical sense. Direct quotes are used to represent the various concepts within each overarching theme; they are not designed to be representative of all concepts within a theme but are aiming to best embody the concepts being portrayed.

### 3.1. Theme 1: Cooperative Discussion

Vets noted that biosecurity discussions and planning were easier when their cattle farmers actively interacted during the consultations. In addition to helping a reciprocal conversation to flow, if cattle farmers could contribute to the biosecurity conversation (or recount some biosecurity information), this indicated to the vet that the farmer understood what was being discussed:

*Vet 4: “[The] Conversation will indicate that he understands the global principles”.*

Furthermore, it emerged that vet–farmer discussions in which both parties were actively involved led to farmers feeling comfortable enough to ask questions during and outside of the consultation.

Vets also deemed “*Cooperative discussion*” to be more effective than simply instructing cattle farmers on biosecurity measures. A reciprocal conversation meant farmers were offering information about the farm, allowing the farmer and vet to “*Get to know each other*” better. Simultaneously, conversational involvement allowed cattle farmers’ own personal input to the development of preventative practices. This suggests that shared decision-making allowed for feasible plans to be jointly constructed, which increased the chances of the recommendations being carried out:

*Vet 5: “Getting him to give himself the answer, I think it’s more powerful that way than standing and lecturing someone”.*

A balanced discussion was repeatedly deemed more effective than a paternalistic consultation. One vet even mentioned that applying the ‘motivational interviewing’ (see further information in the ‘Discussion’ section) technique was a productive method that led cattle farmers to seriously think about the practical implementation of preventative measures, rather than playing a passive role in the conversation:

*Vet 9: “Because they’re not being told what to do and they’re coming up with it themselves then they are more likely to do it”.*

This technique was deemed to result in shared decision-making, assisting in the effectiveness of the biosecurity discussion and subsequent measures that were put into place.

### 3.2. Theme 2: Getting to Know Each Other

The majority of participants reported that, over the course of their working with each other, the relationship with their vet/farmer had become friendly as well as professional. Having numerous interactions over “*Time*” meant that individuals came to know their vet or farmer’s personality and simultaneously learnt a communication style that worked well for them. As a result, vets in particular felt that they could use this to tailor how they discussed biosecurity with the specific cattle farmer, making it much easier to get to the point:

*Vet 4: “[When] you know them better you can be more direct and blunt…whereas some other farmers need examples given”.*

Vets indicated that “getting to know” their cattle farmer allowed them to learn whether their farmer responded best to a direct or indirect communication style and implement this appropriately in order to ensure that conversations regarding biosecurity were effective. This tailoring could be expanded to the types of biosecurity measures that were discussed, as vets reported that they altered the content of their discussions depending on the farmer’s own personal biosecurity values. For example, vets felt that certain farmers took an interest in biosecurity; therefore, it was worth discussing preventative practices in detail in order to plan implementation methods. In contrast, vets learnt that other farmers had no interest in biosecurity generally, or in implementing preventive practices, so biosecurity discussion was pointless:

*Vet 8: “You say it but you know she won’t do it. It’s a bit disheartening but you get used to it…”*

Multiple cattle farmers did express their appreciation for vets who tailored their biosecurity discussion by purposely getting to know the farm and what was feasible for that specific situation. In contrast, one cattle farmer believed that vets who failed to consider the specific features of the farm normally gave very standardised biosecurity instructions, which were often unhelpful and practically unfeasible:

*Cattle farmer 4: “She knows about us and we can’t go isolating individual cows, she understands our system”*

While becoming familiar with each other, it emerged that, over “*Time*”, routine biosecurity discussions developed between some farmers and vets:

*Vet 3: “…he knows us as well. He knows what questions we’re going to ask so he can kind of pre-empt them. But he knows if he gives us a little bit of information first and then we’ll ask the extra bits if we need to. It’s good”.*

Getting to know each other appeared to be beneficial because it meant conversations relating to biosecurity became well-structured and efficient. Therefore, it appears that making an effort regarding “getting to know each other” (including getting to know the farm) is likely essential, not only for the development of efficient communication between vets and cattle farmers, but for biosecurity discussions to become more tailored and individualized to each farm. This demonstrates an overlap between this theme and the theme of “*Time*”.

### 3.3. Theme 3: Time

“*Time*” was repeatedly viewed as imperative by both vets and farmers. “*Time*” was essential for “*Getting to know each other*” and forming good relationships:

*Cattle farmer 6: “[The vet] would come out regularly and we have an excellent relationship with them”.*

Without dedicating “*Time*” to “*Getting to know each other*”, vets would not learn about the individual cattle farmer and the most effective means of communication, or about the mechanisms of the individual farm and the farmer’s biosecurity goals, which impacted the feasibility of preventative practice implementation. As a result, “*Time*” was more important for vets due to the benefits it had on their ability to prescribe feasible biosecurity recommendations.

It was also noted that the development of sufficient biosecurity plans demanded “*Time*” from both the vet and the farmer. Without the “*Time*” to systematically formulate implementation plans, there were no means for the implementation ideas to progress. However, as both cattle-farming and vetting are notoriously busy occupations, mutually available meeting time was generally seen to be restricted:

*Cattle farmer 3: Generally for a farmer to take time out for a vet, they’re not gonna have all the time in the world…there’s just not enough time”.*

Interestingly, cattle farmers rarely had a negative comment to make regarding their vet. When they did, it predominantly related to the lack of time their vet had for them as a paying client:

*Cattle farmer 6: “The only slight criticism I have is their business has expanded really rapidly…I think they’re probably spreading themselves a bit too thin. It’s unavoidable, they want to grow their business, but it would be good to be able to get hold of her a bit more”.*

This quote conceptualises how a cattle farmer who highly values his vet and her advice judges her lack of time to be a barrier in their interactions. Both cattle farmers and vets saw “*Time*” as integral to “*Getting to know each other*” and the elements that coincided with these themes.

### 3.4. Theme 4: Trust

“*Trust*” was identified from interviews with both participant groups and was viewed as a significant factor in vet–farmer relationships and in whether biosecurity advice was implemented by cattle farmers. Without the presence of trust, cattle farmers were unlikely to follow their vets’ advice. However, trust was not automatically given to vets by cattle farmers. Two sub-themes seemed to determine the level at which a farmer trusted their vet’s biosecurity advice. The first sub-theme, “*Farmer experience*”, referred to a farmer’s trust in their vet’s advice, depending on their previous biosecurity experience. In many cases, experienced cattle farmers who had spent decades gaining practical biosecurity knowledge did not always fully trust their vet’s biosecurity advice:

*Cattle farmer 5: “If I have spent 45 years with animals I do pick up a bit of knowledge. A few of the new vets have got it wrong a few times, and I’ve challenged that and said ‘you know, I’m not convinced’…”.*

Cattle farmers utilised their farming experience to evaluate the reliability of their vet’s biosecurity advice. As a result, these farmers did not strictly follow the biosecurity advice from their vet. Conversely, younger farmers who lacked experience in implementing preventative practices were more likely to be receptive to, and trust, their vet’s recommendations without question (regardless of the age of their vet):

*Cattle farmer 1: “I’m quite open minded so if they feel like that’s what needs doing then I’ll listen to them”.*

The second sub-theme was “*Vet experience*”. Experienced cattle farmers who had biosecurity knowledge sometimes had difficulty trusting their vet’s biosecurity advice straight away, especially when the vet was young and viewed to be inexperienced:

*Cattle farmer 8: “Six years ago I probably thought he was a young fool who talks a lot of rubbish, but now he says we should do [a given biosecurity measure] and I would take it on board”.*

This indicates how, over time, cattle farmers learn to trust vets, but the explicit reasons for this gradual trust remain uncertain. It was recognised that, in many cases, a farmer’s trust was gained over “*Time*”, after their vet had had an opportunity to prove their recommended preventative practice to be effective:

*Vet 1: “So once you kind of suggest a few things and you prove that it works, then they’re more inclined to take what you say”.*

In these instances, vets appeared to prove their clinical expertise and that their advice was worth listening to. Therefore, working together over “*Time*” meant the farmer could experience the vet’s ample farming and biosecurity experience first-hand. The cattle farmers were less likely to question their vet’s recommendations going forward, increasing their biosecurity adherence rates:

*Cattle farmer 5: “I think you almost have to earn the respect”.*

Interestingly, two cattle farmers stated that there was a noticeable difference between vets who specialised in farm animals or had previously lived on a farm, and those who also conducted work using other species (equine or small animals). Farmers believed that vets with a stronger farming background had a deeper insight into the mechanisms and practicalities of running a farm, as well as holding greater medical expertise regarding cattle. Both of these benefited the cattle farmer and seemed to contribute to their elevated trust in farm animal vets.

*Cattle farmer 3: “She understands more. She [vet] grew up on a farm so she…has an understanding of where we’re at”.*

Overall, both vets and cattle farmers recognised that the importance of the farmer holding a level of respect and trust in their vet was inherent to whether biosecurity recommendations were undertaken. Yet, vets generally did not necessarily identify that “*Trust*” was important for them within their vet–cattle farmer relationship.

### 3.5. Theme 5: Clarifying the Cost-Effectiveness of Biosecurity Measures

Many farmers implied that the application of a preventative practice largely depended on the cost of putting the biosecurity measures in place, and whether it would save them money in the long-term:

*Cattle farmer 7: “We’re in a business, so anything you can equate to pounds saved. Everything I do is based on cost per litre. So anything that reduces my cost of production…What’s the cost of vaccinating for this disease? It’s going to be X. What is the cost if you have a breakdown with this disease? It’s going to be this…”*

This suggests that the cost-effectiveness of a practice was measured against its disease control abilities once there was already a disease incursion, instead of its disease prevention abilities when there was no immediate threat of disease, which was a stance put forward by many cattle farmers. As with all preventative behaviours, the direct benefit was not always visible. Cattle farmers regularly failed to initially “*Trust*” their new vet’s advice without knowing them very well, and as farmers could not see a benefit to preventative practices, their use was often undermined. Due to this, multiple vets and farmers vocalised the benefits of presenting farmers with monetary statistics where the expenditure and savings of a preventative practice were clearly outlined:

*Vet 7: “…having some useful stats [statistics] to hand because it’s something that I tend to discuss in quite broad terms, but having some numbers instantly to hand that you can just rattle off might give more meaning…I think having some instant numbers to hand would be beneficial”.*

This demonstrates how instead of having a generalised discussion around biosecurity, in which the cattle farmer had to “*Trust*” the vet’s recommendations, statistics assisted farmers in comprehending the economic details of preventative practice implementation and allowed them to more easily identify the benefits. Alongside making the cost-effectiveness of measures instantly clearer for farmers, the breakdown of monetary spending means that this technique also provides evidence to support a vet’s biosecurity recommendations, which simultaneously adds credibility to, and increases “*Trust*” in, their biosecurity advice:

*Vet 8: “[using this technique] they can see a monetary gain…. I think it’s harder to have those conversations if they aren’t seeing a direct benefit to it”.*

Vets also consistently reported that their cattle farmers became more enthusiastic about preventative practices once they experienced their own disease breakdown, which required them to pay a large sum of money unexpectedly to cover disease costs. In these cases, cattle farmers had ignored their vet’s advice and failed to consider the long-term cost-benefit of preventative practices, simply because the measures were not perceived to be cost-advantageous. However, when large monetary losses were incurred, this seemed to motivate the farmer to become proactive in biosecurity measures, instead of reactive:

*Vet 1: “It wasn’t until he actually had it with cattle dropping dead in front of his eyes that he thought it was a proper problem [biosecurity] for him…now he’s a complete convert”.*

In the singular case where a vet’s farmer was not carrying out good levels of biosecurity, the vet theorised that the reason behind this was that the farmer rented the land on which he reared his animals, meaning that no biosecurity measure would serve the cattle farmer in the long-term and, therefore, these measures lacked cost-effectiveness.

This recurring pattern of cattle farmers paying more attention to biosecurity measures after personally experiencing disease breakdown themselves adds to the implication that farmers holding an explicit understanding of the long-term cost-effectiveness of biosecurity measures increases the likelihood of them implementing these preventative practices.

## 4. Discussion

This exploratory study identified five main themes within the vet–cattle farmer relationship that appear to be influential on whether biosecurity measures are carried out on farms. These are as follows: the vet–farmer getting to know each other, which allows for tailored communicative styles and individualised biosecurity discussions, trust in the vet’s advice, understanding of the cost-effectiveness of biosecurity measures, cooperative biosecurity discussions that incorporate shared decision-making, and finally, the amount of time dedicated to biosecurity discussions. Each of these often-interlinking factors aligned with cattle farmers who were carrying out good levels of biosecurity on farms. This key information, gleaned from interviewing vets and cattle farmers from across England, is useful for those currently in the field, allowing them to potentially adjust their communication approach and manage expectations before and during discussions, and is useful for integration into training for those who will be in the field in the future (e.g., veterinary and agricultural undergraduate and postgraduate students).

“*Getting to know each other*” allowed for vets in particular to tailor their communication style and the content of the biosecurity discussions, assisting cattle farmers in understanding the biosecurity information they receive, which then allows for biosecurity measures to be developed in relation to the farmer’s biosecurity goals and abilities. This mirrors previous vet–farmer research, which showed that vets recognised that using different communicative techniques enhanced message effectiveness across different farmers [19]. In human medicine, patient satisfaction and medical adherence were found to be relative to the degree of tailored communication applied by the clinician during the consultation [35]. However, the fact that vets within this study considered the biosecurity values of their farmer to judge the feasibility of biosecurity measures contrasts with previous work by Bard, Main, Haase, Whay, Roe and Reyher [21], who reported that vets often fail to understand their farmer’s biosecurity goals. This discrepancy could be due to the fact that cattle farmers included in the present study were believed to be carrying out high levels of biosecurity practice, versus those reportedly maintaining broken biosecurity efforts in Bard, Main, Haase, Whay, Roe and Reyher [21]. The contrast between these two studies contributes to the argument that shared goals between vets and farmers are fundamental to a successful biosecurity discussion [21], increasing the chance of effective preventative practices being put into place [18,36,37].

Not only was “*Time*” essential for “*Getting to know each other*”, it also led to biosecurity discussions becoming habitual routines in some cases. “*Time*” was consistently highlighted by vets and cattle farmers as a substantial barrier to their biosecurity conversations, especially from the cattle farmer’s perspective. Yet, a routine conversation between vets and farmers who are already familiar with each other and the farm’s context to some extent overcame these restrictions of “*Time*”. Lack of time emerges throughout the human healthcare communication literature, as reports show that doctors attempting to share information to implement preventative healthcare are restricted [38], and that time limitations also inhibit effective communication [39] and the establishing of strong doctor–patient relationships [40]. Veterinary clients who perceive their consultation as too short are less likely to comply with clinical recommendations [41], perhaps because the importance of the recommendation cannot be emphasised sufficiently within a restricted timeframe. Therefore, a lack of time can have negative consequences for both human patients and, in this study, cattle farmers attempting to carry out preventative practices. Thus, dedicating *“Time”* to biosecurity discussions benefited “*Getting to know each other*”, and vice versa, because vets and cattle farmers who repeatedly have similar biosecurity discussions increase their familiarity with the conversation structure, allowing for them to pre-empt conversations. Simultaneously, this allows for the discussion to run methodically, which can overcome the restrictions of “*Time*” [15].

Our findings demonstrate that the level of trust a cattle farmer has in their vet depended on, firstly, the individual “*Farmer experience*” of biosecurity and, secondly, “*Vet experience*” in farming and biosecurity. Specifically, when vets were viewed as young or inexperienced, older cattle farmers believed their own practical experience of biosecurity to be more valuable than their vet’s, and so biosecurity advice was not followed. This links with previous research, as Higgins et al. [42] and Brocket et al. [43] revealed how clients tended to trust senior vets more than newly graduated ones. Farmers hold experience in the practical implementation of biosecurity on their singular farm, whilst vets practice across numerous farms and implement unique programmes, as well as holding theoretical knowledge from their training. Ultimately, knowledge from both stakeholders is important in order to formulate effective biosecurity measures relevant for an individual farm [19].

It was also found that vets who had a farming background, or who had specialised expertise in large animals, were trusted more by cattle farmers. In order to overcome this, a farmer’s trust could be gained when a vet’s previous recommendations prove effective, demonstrating expertise, meaning that farmers will trust the vet’s advice going forward. Overall, it was deemed that the experience level of the vet and cattle farmer predicted the farmer’s immediate level of trust in their vet, but trust could be increased by vets proving themselves to farmers over time, which has also been shown in other studies [44]. Although it is continually recognised throughout human healthcare contexts that trust in a clinician is important for client compliance [45,46], the notion of a medical expert needing to “prove” themselves as trustworthy may be exclusive to vets. Doctor–patient relationships assume “institutional trust” [47], and currently there is no evidence exhibiting that patients trust older physicians more than younger physicians. Yet, this study, alongside previous evidence, indicates that vet–cattle farmer relationships generally assume “secure trust” [48], in which repeated encounters (where the vet’s advice is tested and proven to be effective) allow for trust to build gradually over time*,* which coincides with “*Getting to know each other*”. The difference between doctor-patient relationships holding “institutional trust” and vet–farmer relationships maintaining “secure trust” may be due to the fact that doctors serve patients, who are generally valued more than animals. In comparison, a vet’s medical advice is likely to also hold economic value to farmers [20]. As this is an important concern for farmers, they will seek confirmation that the suggested medical recommendations are cost-effective before implementing them [2,49]. This may be why “*Clarifying cost-effectiveness*” is essential in convincing cattle farmers to implement biosecurity measures [4]. Here, “*Clarifying cost-effectiveness*” is an additional mediating factor for gaining a farmer’s trust.

Irrespective of trusting a vet’s advice, the importance of “*Clarifying cost-effectiveness of biosecurity measures*” became apparent, as understanding the economic advantage of preventing disease (rather than controlling it once it becomes an issue) led cattle farmers to take biosecurity seriously. For example, one vet reported that their cattle farmer carried out minimal levels of biosecurity because they were temporarily renting the land. Therefore, the farmer did not face any long-term biosecurity risks, and the short-term risks were not sufficient to justify putting measures in place from which a temporary benefit would be gained from, and alongside, a substantial pay-out. This supports the notion that comprehending the long-term monetary savings provided by preventative measures motivates farmers to implement biosecurity measures [4,50]. Vets reported that the long-term benefits and cost-effectiveness of biosecurity measures were often difficult to portray to cattle farmers, especially when farmers could not visibly see the immediate effects of a measure, and therefore would not “*Trust*” the vets’ advice. However, multiple participants suggested that having specific figures detailing the initial monetary outlay alongside the long-term monetary savings from the biosecurity measure would explicitly determine its effectiveness and whether it was worth implementing. This notion of “weighing up” a method’s effectiveness before implementing it is reflected across human healthcare contexts within the popular “Necessity-Concerns Framework” (NCF) [51]. This NCF approach postulates that, upon being prescribed medication by one’s physician, patients often weigh up concerns about the medication alongside beliefs in its necessity; it is more likely that patients will adhere to the medication if their belief that the treatment is needed outweighs their concern about taking the medication. This concordance between adherence rates depending on the cost-effectiveness/necessity–concerns demonstrates another similarity between human and farm animal healthcare.

*“Cooperative discussion*” in veterinary consultations appears to be critical for vet–cattle farmer relationships. Without farmers fully comprehending biosecurity information, implementation is unlikely to be accurate [19] and, therefore, ineffective. Additionally, a farmer actively engaging in conversation with their vet allows for shared decision-making in the planning of biosecurity measures specific to that farm [37,52]. This also links to the theme of *“Getting to know each other*” because an actively involved cattle farmer also offers information about the farm to their vet, which allows for the vet to tailor biosecurity to the specific farm in question. As a consequence, individualised biosecurity measures are formulated between the pair [36], increasing the farmer’s adherence levels [15,53]. Previous medical research has determined the substantiality of a balanced clinical discussion in which clients can voice their own concerns and have personal input into the formulation of medical treatment plans, leading to elevated levels of medical adherence [54].

The significance of client engagement within medical discussions gives way to the technique of ‘motivational interviewing’ (MI) [55]. As one vet stated, MI promotes client’s autonomous decision-making by prompting their own thoughts on how they might personally alter their farm to prevent pathogen transmission. Motivational interviewing ensures that treatment plans are relevant to the individual’s health values and not generalised medical recommendations [55]. Although the MI technique requires training of the medical professional, emerging evidence certainly supports the effectiveness of its application [56,57]. For example, a systematic review by Rubak, et al. [58] showed that, in 80% of included studies, MI surpassed the effectiveness of traditional advice-giving in human healthcare. Considering the fact that MI holds ample empirical support and incorporates elements of healthcare communication that are proven to increase patient adherence rates, its application would expectedly improve the vet–farmer biosecurity discussions more than alternative communicative techniques currently used within the veterinary field, such as the central and peripheral communication routes [22]. The central route suggests the use of direct science-based information with farmers who are already motivated, whereas the peripheral route suggests the application of heuristics and persuasive techniques with farmers who take less interest in biosecurity. In addition, MI and the dual-route model both incorporate similar factors deemed important within healthcare communication, such as individualised goal setting, personalised treatment-planning and shared decision-making [22]. However, the prevailing success of MI studies indicates that MI applies these factors in a more effective manner than the central and peripheral route strategy. Other approaches are used, such as the Transtheoretical Model of Change (Stages of Change Theory) [59], cognitive therapy or Relational therapy, which could be applied to these scenarios in future studies to explore the potential that they will help to improve biosecurity uptake [60].

### 4.1. Study Limitations

Although this study identified several factors within a vet–cattle farmer relationship that may influence biosecurity on farms, we should acknowledge that there are many other factors that will also impact this, which have been reported previously [61]. For example, a farmer’s expenditure allowance will largely impact the complexity of the preventative practices undertaken out on his or her farm [2]. However, these factors were not the focus of this research project.

A convenience sample approach was taken here, including inviting farmers who were involved in previous research on biosecurity to take part, which could have resulted in different findings than if farmers not involved with previous research had been approached. Furthermore, we relied on self-reports of farmers’ biosecurity levels. Self-reports can be influenced by the social desirability effect [62]: participants may be embarrassed to report poor levels of biosecurity. As a result, farmers may have claimed that they carried out sufficient levels of biosecurity when this was not the case. However, it is unlikely that participants would have agreed to take part in a study investigating biosecurity if they were not putting in place reasonable preventative practices.

### 4.2. Future Research

Our findings have highlighted key areas for both large animal vets and cattle farmers to prioritise within their relationship and their biosecurity discussions. Key factors such as “*Clarifying cost-effectiveness of biosecurity measures*”, could be included in consultations. How easy this would be for vets to consistently deliver would require further investigation [63]. Other factors, including *“Time”*, may be hard to address due to the complexity of this topic (not enough time, time to focus on biosecurity specifically, etc.). However, it has been suggested that improving communication by “*Getting to know each other*” can assist in this restriction.

The growing evidence of the use of MI in the veterinary context [23,26,64] is encouraging, though further research is needed to consolidate its efficacy. The small number of vet–farmer studies in this area does not provide the same volume of evidence that MI does within the human healthcare literature.

## 5. Conclusions

The overall results of this exploratory interview-based study indicate that there are important interlinking factors within a vet–cattle farmer relationship that impact the biosecurity measures being undertaken on farms in England: how well the vet–cattle farmer know each other, degree of cooperative discussion, the level of trust held, clarification of cost-effectiveness of biosecurity measures and, importantly, the amount of time given to biosecurity discussions. Ultimately, for successful implementation in practice, these factors must be acknowledged, the degree to which they are likely to hinder progress assessed, and adjustments made accordingly.

## Data Availability

Due to the confidential nature of the vet–client relationship and the subsequent questions asked in this study, interview respondents were informed that their data would remain confidential and would not be shared.

## Supplementary Materials

The following supporting information can be downloaded at: https://www.mdpi.com/article/10.3390/vetsci10070410/s1.
