# Supplementary material for: Factors within A Veterinarian-Cattle Farmer Relationship That May Impact on Biosecurity Being Carried out on Farms: An Exploratory Study
Source: Vet Sci. 2023 Jun 23;10(7):410. doi: 10.3390/vetsci10070410 (PMC10383729; doi:10.3390/vetsci10070410)
Supplement: Supplementary file 1 [file vetsci-10-00410-s001.zip › vetsci-2400202-supplementary.pdf]

Supplementary Materials: Interview guides used for farmers and vets

Introduction:

Hi, my name is xxxx. Studying xxx at xxxxxxx.
Wanted to start by saying how grateful I am for you to take time out of your day to talk to me, really appreciate it as know you are so busy.
Research I am doing is very important because there is a severe lack of research into vet-farmer relationships and how this influences biosecurity on farms. Important for health of animals and to make sure you get the most from your consultations with the vet.
Will start by asking about your experience and time working as a farmer/vet before asking about your relationship with your vet/farmer, and your discussions around biosecurity, so please tell me as much information as you feel comfortable with.
Just to confirm you have read and signed the consent form and happy for everything to go ahead?

Farmers Questions:

1. Whereabouts in England is your farm?
2. Can I ask how old you are?

(Gender? [*don't actually ask this*])

3. How long have you been a farmer for?
4. How many cattle do you own at this time?
5. Do you have any farm or agricultural qualifications?
6. Are you a member of any farm assurance schemes?
7. Who is your vet?

(Do they visit for routine visits/what type of vet?)

8. How often do they visit your farm?

I'm interested to hear about your relationship with your vet:

9. How would you describe your relationship with your vet?

(Good/bad/friendly/professional/comfortable/avoided)

10. What do you like about your vet?

(Friendly/professional/treatment of animals/caring/easy to access)

11. And what do you like least about your vet?

(Unfriendly/unprofessional/uncaring)

12. Would you say you tend to follow the advice of your vet?

(How much?)

13. Has your relationship changed with your vet over the years?

(How? More friendly/professional/understand each other better)

Regarding your actual consultations with your vet:

14. Who leads the discussion during your discussion when the vet comes to the farm, and do you like it that way?

(Is it split 50/50? What percentage?)

15. Are you given an opportunity to clarify information you are unsure about, or ask further questions, during your vet's visit?

I'm now going to ask you some questions about biosecurity:

16. How would you describe biosecurity?

17. Can you give any examples of biosecurity measures you carry out on your farm?
18. Does your vet discuss biosecurity with you?
(If no, continue to question 19. If yes, move to question 22).

19. Would you like to be given the chance to discuss and plan biosecurity measures during the discussions?
20. Why do you think your vet does not discuss biosecurity measures with you?
(Already satisfactory/vet not interested in biosecurity/lazy/inexperienced)
21. Do you feel your current preventative practice is effective enough without your vet's input?
(Completely or like a little input)?

So just a few questions regarding the nature of these biosecurity discussions:
22. What kind of biosecurity aspects are discussed?
23. Can you remember the last time you discussed biosecurity with this vet? Tell me about the discussion you had with them
(Conversation a matter of minutes, hours? Were they helpful?)
24. Do you think these types of conversations are important?
25. Does your vet understand your biosecurity goals?
(Share the same goals as you?)
26. Are the vet's expectations in relation to the biosecurity you carry out on your farm feasible?
27. What does your/could your vet do to motivate you to carry out biosecurity measures?
28. Do you/would you trust your vet's advice on biosecurity?

And more generally:
29. Is there anything your vet could do to make the discussion about biosecurity better in your eyes?
30. Is there anything else you would like to comment on your discussions with your vet?

Thank you very much for speaking to me today, do you have any questions?

Veterinary Questions:

1. Whereabouts are you based? How long have you been a vet for?
2. How often do you visit the farmer you have in your mind?
3. Is this someone you like seeing?
4. How long have you been their vet for?
5. And can I ask how and when did you qualify as vet?

I'm interested to find out about your relationship with your/this cattle farmer:
6. How would you describe your relationship with this farmer?
(Good/bad/friendly/professional/comfortable/avoided)
7. Has your relationship changed with this farmer over the years?
(How? More friendly/professional/understand each other better)

Regarding your actual consultations with your farmer:
8. Who leads the discussion during the visit with this particular farmer?
(Do you like it that way?)
9. What would you say the percentage split would be between you in terms of who leads the discussion?
10. Do you give this farmer an opportunity to ask you questions?
11. Is there anything you think that yourself or this farmer could do to make the communication better between you?

Now some questions about biosecurity:
12. How would you describe biosecurity?
13. Can you think of examples that demonstrate a high level of biosecurity?
14. Do you discuss biosecurity with this farmer?
15. If yes to question 14, what would you judge their level of biosecurity to be at? (Why?). Now move to question 18.
If no to question 14, continue to question 16.
16. Why do you not discuss biosecurity with your farmer?
(Farmer not interested/already satisfactory levels/you are not interested/lack of experience)
17. Do you think their preventative practice is sufficient enough without your advice? (Need a lot of help/little help).

18. What aspects of biosecurity do you discuss with this farmer?
19. Can you remember the last time you discussed biosecurity with this farmer? Can you tell me about the discussion you had with them?
(Conversation a matter of minutes, hours? Who led conversation? Were they disinterested or enthusiastic?)
20. Do you think these types of conversations are important?
21. Do you think your farmer wants to be actively involved in planning future biosecurity measures?
22. How do you/would you know if the farmer has fully understood the biosecurity measures being discussed?
23. How would you/have you previously motivated your farmer to ensure the best possible biosecurity measures are carried out?
24. Does your/would your farmer trust the biosecurity advice you gave them?
25. Is there anything you think that yourself or the farmer could do to make the discussion around biosecurity more effective?
26. Is there anything else you would like to comment on regarding your discussions with this farmer?

Thank you very much for speaking to me today, do you have any questions?
